# Efficacy and safety of using a unilateral lower limb exoskeleton combined with conventional treatment in post-stroke rehabilitation: a randomized controlled trial

**DOI:** 10.3389/fbioe.2024.1441986

**Published:** 2024-09-18

**Authors:** Ying Jin, Bing Xiong, Lina Chen, Weiwei Zhao, Zhe Li, Chi Zhang, Xin Xu

**Affiliations:** ^1^ Department of Rehabilitation in Traditional Chinese Medicine, The Second Affiliated Hospital of Zhejiang University School of Medicine, Hangzhou, China; ^2^ Hangzhou First People’s Hospital, The Forth School of Clinical Medicine, Zhejiang Chinese Medical University, Hangzhou, China; ^3^ The First Hospital of Jiaxing, The Affiliated Hospital of Jiaxing University, Jiaxing, China; ^4^ The Fifth Affiliated Hospital, School of Medicine, Zhengzhou University, Zhengzhou, China

**Keywords:** exoskeleton, hemiplegia, rehabilitation, balance function, post-stroke

## Abstract

**Introduction:**

The incidence of hemiplegia caused by stroke is high. In particular, lower limb dysfunction affects the daily activities of patients, and lower limb robotic devices have been proposed to provide rehabilitation therapy to improve balance function in this patient population.

**Objective:**

To assess the effectiveness of the LiteStepper^®^ unilateral lower limb exoskeleton (ULLE) combined with conventional treatment for balance function training in patients with post-stroke hemiplegia.

**Methods:**

This multicenter randomized controlled trial, conducted in the convalescent rehabilitation ward of four hospitals, involved 92 patients in their post-stroke phase. Participants were randomized into an experimental group (EG) or a conventional group (CG). The EG adopted the LiteStepper^®^ ULLE combined with conventional treatment for 21 days. The CG underwent a standard daily rehabilitation routine for 21 days. The Berg Balance Scale (BBS), Functional Ambulation Category scale (FAC), 6-min walk test (6MWT), and Barthel Index (Barthel) were used for evaluations before and after 21 days of rehabilitative training.

**Results:**

The BBS scores in EG was significantly elevated compared to CG, exhibiting a profound statistical difference (*P<* 0.0001). Notably, these disparities persisted at both day 21 (*P <* 0.0001) and day 14 (*P <* 0.0047) post-intervention, underscoring the efficacy of the treatment in the EG. The EG demonstrated a markedly greater improvement in BBS scores from pre-rehabilitation to 21 days post-training, significantly outperforming the CG. Furthermore, at both day 14 and day 21, functional assessments including the FAC, 6MWT, and Barthel revealed improvements in both groups. However, the improvements in the EG were statistically significant compared to the CG at both time points: day 14 (FAC, *P =* 0.0377; 6MWT, *P* = 0.0494; Barthel, *P* = 0.0225) and day 21 (FAC, *P* = 0.0015; 6MWT, *P* = 0.0005; Barthel, *P* = 0.0004). These findings highlight the superiority of the intervention in the EG in enhancing functional outcomes. Regarding safety, the analysis revealed a solitary adverse event (AEs) related to the LiteStepper®ULLE device during the study period, affirming the combination therapy’s safety profile when administered alongside conventional balance training in post-stroke hemiplegic patients. This underscores the feasibility and potential of incorporating LiteStepper®ULLE into rehabilitation protocols for this patient population.

**Discussion and significance:**

The LiteStepper^®^ ULLE combined with conventional treatment is effective and safe for balance function training in patients with post-stroke hemiplegia.

## 1 Introduction

The incidence of stroke is increasing annually, and it is expected that by 2030, the global age-standardized incidence of stroke will increase to 89.32 per 1,00,000 population (Pu et al., 2023). Following severe stroke, many patients die, and those who survive may have a greatly reduced quality of life due to sequelae such as hemiplegia. More than half of the patients with stroke may develop hemiplegia (Alhwoaimel et al., 2024). In such patients, a lot of time and energy is often required for their limb function to recover; therefore, a variety of rehabilitation techniques to restore the limb function of patients with hemiplegia have been explored (Li et al., 2024). At present, the conventional rehabilitation treatment for hemiplegia after stroke is symptomatic treatment (Wang et al., 2023), including motor imagination therapy, compulsory motor therapy, and robot-assisted therapy (Lee et al., 2022; Loro et al., 2023). Sensory function rehabilitation through transcranial magnetic stimulation and cranial electrical stimulation to stimulate neuronal activity has also been implemented (Karatzetzou et al., 2022). The above rehabilitation methods have certain functional rehabilitation effects. However, the rehabilitation of sensory and motor functions is often not conducted simultaneously, and even though most patients with hemiplegia are eager to recover, it is easy to develop circular gait, and balance function and gait symmetry remain poor (Warutkar et al., 2022). The long recovery time of patients diminishes their enthusiasm for life and aggravates social and economic pressure. Therefore, it is imperative to explore efficient rehabilitation methods.

In clinical practice, robot-assisted therapy has been proven to be an effective means of improving balance function (Takebayashi et al., 2022). Current traditional bilateral lower limb exoskeleton rehabilitation robots are relatively heavy, and the constrained posture can cause patients to lose balance or even fall. This restriction on the healthy side is not conducive to the deep relearning of movements by patients (Qin et al., 2023). Moreover, the passive rehabilitation training for both the healthy and affected sides is not beneficial for the control of the rehabilitation exoskeleton. Exoskeletons typically operate on predetermined motion paths suitable for assisting specific limbs or body parts, but these paths are not personalized for each patient, as they are relatively fixed (Hui et al., 2024). If the motion path suitable for the patient can be monitored to achieve more natural and intuitive interaction between the patient and the exoskeleton device, the rehabilitation efficiency can be improved (Jyotindra Narayan et al., 2023).

Studies have shown that based on the theory of neuroplasticity, mirror neuron rehabilitation and motor imagery therapy contribute to the regeneration, reorganization, and remodeling of neural functions affected by brain injury (Joy and Carmichael, 2021; Abdullahi et al., 2022). These rehabilitation treatment methods are both clinically significant and effective for the rehabilitation of the central nervous system (CNS). However, the current rehabilitation methods stimulate the sensory pathways of the peripheral nervous system (PNS) while neglecting the rehabilitation of the motor pathways (O'Dell, 2023); this is not conducive to the patient’s motor relearning program (Zhang et al., 2020). Furthermore, it is difficult to rehabilitate both sensory and motor pathways in patients in clinical practice. Nevertheless, complete neurological rehabilitation may benefit from closed-loop training of the CNS and PNS (including motor and sensory pathways) (Aze et al., 2023). This requires the active participation of the patient.

To overcome these challenges associated with robot-assisted therapy, LiteStepper^®^ unilateral lower limb exoskeleton (ULLE) was developed by Angelexo Science Co., LTD., Zhejiang, China based on symmetrical gait control, as a new type of lower limb rehabilitation training equipment for patients with hemiplegia after stroke. The ULLE design requires the patient to wear a sensing device on the healthy side that senses the movement of the limb based on physiology, kinematics, dynamics, and mechanics. The affected side wears the power unit combined with the intelligent learning advantages of the exoskeleton rehabilitation robot. This approach is more conducive to stimulating the patient’s active learning ability from the healthy side to the affected side, thereby helping the patient recover balance function and perform normal gait (Çalıkuşu et al., 2022). Since ULLE has not been widely used for balance function training, limited data exist regarding its effectiveness and safety for patients with hemiplegia after stroke. Therefore, we aimed to evaluate the effectiveness and safety of LiteStepper^®^ ULLE combined with conventional treatment for balance function training in patients with post-stroke hemiplegia to facilitate its clinical application.

The main objective of the study was to assess balance function-related parameters while using LiteStepper^®^ ULLE, such as sitting balance, standing balance, and dynamic balance, as assessed by the BBS. The secondary objective was to assess changes in walking ability and activities of daily living during recovery with LiteStepper^®^ ULLE, quantified by the FAC, 6MWT, and Barthel Barthel, which were used to evaluate the effectiveness of the device. We also assessed AEs during the use of LiteStepper^®^ ULLE to evaluate its safety.

## 2 Materials and methods

This multicenter, randomized controlled, open trial, conducted over 21 days, included 92 patients with post-stroke hemiplegia enrolled in rehabilitation between 23 November 2021 and 20 June 2022. Four centers participated in this study, and Table 1 shows the assignment of clinical trial cases in the four centers. To ensure randomization, this experiment used the concealed envelope method; participants were randomly assigned to the experimental group (EG) or conventional group (CG) in a ratio of 1:1. After confirming the patients’ eligibility, the researchers opened the corresponding random envelopes for randomization to reduce subjective factors when selecting patients. Due to the different treatment methods between the EG and CG, blinding of the researchers and patients was not possible.

**TABLE 1:** Assignment of cases at the four centers.

Centers	Clinical research institution	Total number of cases	Number of cases in the experimental group (n)	Number of cases in the conventional group (n)
01	Hospital 1	24	12	12
02	Hospital 2	52	26	26
03	Hospital 3	8	4	4
04	Hospital 4	8	4	4

### 2.1 Protocol

All included patients received conventional treatment. The CG included patients with post-stroke hemiplegia who received conventional treatment. The EG included patients with post-stroke hemiplegia who on the basis of conventional treatment adopted the LiteStepper^®^ ULLE for rehabilitation management.

The conventional treatment included two parts, lasting 60 min in total. One part was functional electrical stimulation, performed once daily for 20 min per session; the other part was comprehensive training, performed once daily for 40 min per session and consisting of neuromuscular facilitation techniques, hip and knee joint control training, ankle dorsiflexion-induced training, affected lower limb support training, balance training (sitting, standing), and ambulation training.

Following conventional treatment and at least 2 h of rest, the EG received the experimental treatment, with each 40-min session consisting of 10 min of passive stretching followed by 20 min of walking training with LiteStepper^®^ ULLE and a final 10-min phase of passive stretching. The walking speed was mainly tolerated by the patients. The trial had an open design; the researchers and participants were aware of the treatment allocation.

All of the treatments were performed on the ground. Over the 21-day therapeutic intervention, two groups were evaluated for comprehensive balance, walking ability, and AEs.

### 2.2 Ethical considerations

Overall, 92 patients in their post-stroke phase participated in this study. The protocol was conducted in accordance with the principles of the Declaration of Helsinki and was approved by the appropriate Ethics Review Committee (The Second Affiliated Hospital of Zhejiang University School of Medicine, permission number: 2021-0323; Hangzhou First People’s Hospital, permission number: 2021-08001; The First Hospital of Jiaxing, permission number: 2021-025; The Fifth Affiliated Hospital, permission number: 2022-02-005-K02). All participants provided written informed consent before participation. The Consolidated Standards of Reporting Trials reporting guideline was adhered to when designing and reporting this trial. This trial was prospectively registered at ClinicalTrials.gov (identifier: NCT05360017).

The inclusion criteria were as follows:

1) understanding the entire experimental process, voluntarily participating, and signing the informed consent form;
2) having an age of 18–75 years and being of either sex;
3) having stable vital signs, stable condition, and tolerance to low-intensity sitting and standing training;
4) having a body mass of ≤100 kg and a height of 1.50–1.90 m;
5) having post-stroke hemiplegia, including cerebral infarction and intracerebral hemorrhage, diagnosed at the first onset, and disease course was within 1 month after its onset; and
6) having passive range of motion of the hip and knee that was not significantly limited and whose passive range of motion of the ankle could be maintained in a neutral position.

The exclusion criteria were as follows:

1) severely limited range of motion of the joint and limited walking movement;
2) skin injury or infection of the lower limb in contact with the robot;
3) history of unstable angina pectoris, severe arrhythmia, and other heart diseases;
4) history of severe chronic obstructive pulmonary disease;
5) other contraindications or complications that may affect walking training;
6) pregnant women and women preparing for pregnancy or lactation;
7) participation in any clinical trial within 1 month before enrollment in this study; and
8) other conditions that the researcher deemed ineligible for this clinical trial.

### 2.3 Description of the equipment

**FIGURE 1:**
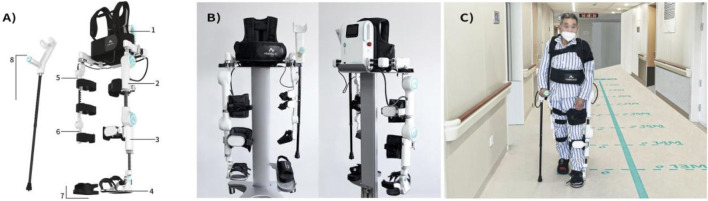
Structural components and patient usage diagram of LiteStepper: **(A)** Mechanical components: 1-system host backpack; affected module including 2-affected thigh, 3-affected calf, 4-affected shoe; unaffected module including 5-unaffected thigh, 6-unaffected calf, 7-unaffected shoe; and 8-elbow crutch (with handle); **(B)** front and rear views of LiteStepper; **(C)** the patient usage of LiteStepper.

The specifications of the LiteStepper^®^ ULLE exoskeleton are as follows:

1) Weight: The LiteStepper^®^ ULLE weighs 16.9 kg, making it a relatively lightweight exoskeleton designed for ease of use and patient comfort during rehabilitation sessions.
2) Dimensions: When fully deployed, the LiteStepper^®^ ULLE measures 1,460 mm in length, 520 mm in width, and 480 mm in height. These dimensions accommodate a wide range of patient body sizes and allow for ample natural movement during rehabilitation exercises. For transport and storage, the device can be compacted to a size of 1,310 mm in length, 600 mm in width, and 380 mm in height.
3) Power Source: The device is powered by an independent 37 V, 2900 mAh battery, with an option to include two batteries for rapid replacement during extended use or training sessions. This ensures that the device remains operational for extended periods without a constant power source, making it suitable for various rehabilitation settings.
4) Charging: The battery can be recharged using a charger with specifications of 220 V, 50 Hz input and 42 V, 2 A output, ensuring efficient and safe charging for uninterrupted use of the LiteStepper^®^ ULLE.
5) Sensors: The LiteStepper^®^ ULLE incorporates advanced sensor technology to monitor and adjust its assistance based on the patient’s movements and needs. The sensors include:
  ① Graphene Pressure Sensors: These are strategically located, such as in the unaffected shoe, to provide real-time feedback on pressure distribution during walking and standing exercises.
  ② Absolute Angle Sensors: Positioned at the hip and knee joints of the unaffected leg, these sensors capture gait information and movement intention, enabling coordinated movements between the affected and unaffected sides.
  ③ Cane Pressure Sensor: Integrated into the bottom of the assistive elbow crutch, this sensor detects the user’s weight distribution on the cane, providing additional stability and safety during training.
6) Motor Specifications: The LiteStepper^®^ ULLE is equipped with brushless servo motors capable of delivering a control torque of 45 Nm with an operating voltage range of 36-42 V. These motors feature a high gear ratio of 100:1, resulting in a maximum output power of 147 W and a peak torque of 88.63 Nm. The motors communicate via an isolated CAN bus, ensuring reliable and efficient data transmission (Figure 1).

LiteStepper has two different training modes:

Training Mode I: When the patient is in training initially, the unaffected module captures the motion data on the patient’s unaffected side, including parameters such as step length, step height, and step speed; the unaffected shoe provides pressure feedback; the assistive elbow crutch supports body balance; and the trigger button on the crutch handle can be operated to initiate the walk of the affected side with the exoskeleton. The system host processes data through data analysis to ensure that LiteStepper can help the patient complete the walking process according to the gait parameters obtained from the unaffected side.
Training mode II: When the patient is in training and can effectively handle walking with the exoskeleton, the unaffected module captures the motion data on the patient’s unaffected side, including parameters such as step length, step height, and step speed; the unaffected shoe provides pressure feedback; the assistive elbow crutch supports body balance; and the exoskeleton is triggered automatically without pressing the trigger button on the crutch handle to assist the patient in walking more naturally. The system host processes data through data analysis to ensure that LiteStepper can help the patient complete the walking process according to the gait parameters obtained from the unaffected side.

### 2.4 Balance and walking ability assessment

The first condition for normal walking is to maintain balance. Balance is a key factor that helps patients with post-stroke hemiplegia achieve optimal gait function.

The BBS is reliable, valid, and widely used in clinical practice. It can be administered easily with minimal equipment in 10–15 min; more so, it can be applied to people who are unable to move from a chair (Baronchelli et al., 2021). The BBS comprises 14 items: one item for sitting balance (task 1: sitting unsupported); eight items for standing balance (task 2: standing unsupported, task 3: standing with eyes closed, task 4: standing with feet together, task 5: standing on one foot, task 6: turning to look behind, task 7: grab an object from the floor, task 8: reaching forward with outstretched arms while standing, and task 9: placing one foot in front of the other); and five items for dynamic balance (task 10: going from sitting to standing, task 11: going from standing to sitting, task 12: transfer from a seat with an armrest to a seat without an armrest, task 13: turn 360°, and task 14: place alternating foot on a step or stool while standing unsupported).

The total score ranges from 0 (severe) to 56 (mild) points, and the fall risk cutoff score is less than 40.

To increase the reliability of the evaluation results, we also recorded the changes in the FAC, 6 MWT, and Barthel when patients were treated with the device; this is a secondary way to assess the effectiveness of the device in rehabilitating the affected limb (Van Criekinge et al., 2024).

The FAC is an ordinal scale used to classify the level of support required for walking safely, whether using lower limb orthoses or walking aids (Moore et al., 2020). It has shown good reliability, and its structural validity has been confirmed. Furthermore, it can also be applied to individuals who are unable to walk independently. The FAC comprises six items and ranges from 0 (unable to walk without the assistance of two people) to 5 (independent walking on uneven surfaces and on stairs).

The 6 MWT is an assessment of functional capacity and measures the maximum walking distance of participants within 6 min (Jung and Oh, 2023). It is superior to other walking tests in that it is safe and easy to administer.

Barthel has good test-retest reliability and validity in patients with post-stroke hemiplegia (Ocagli et al., 2021). Barthel encompasses 10 items and assesses independence in activities of daily living. Its maximum score is 100 points, with lower scores indicating a greater need for help and support to complete each activity.

### 2.5 Data analysis

Data analysis was performed from 20 June 2022 to 20 July 2022. All data were analyzed using IBM SPSS Statistics, version 25.0 (IBM Corp., Armonk, NY, USA). Statistical significance was set at *P* < 0.05. The Shapiro–Wilk test was performed, and histograms, as well as P-P and Q-Q plots, were interpreted to confirm whether the data were normally distributed. Normally distributed data are presented as the mean ± standard deviation and were analyzed using the independent sample t-test. Non-normally distributed data are presented as the medium (25th percentile, 75th percentile) and were analyzed using the Wilcoxon signed-rank test. The EG treatment was considered superior to the CG treatment if the BBS before and after 21 days of rehabilitation training was greater than the superiority margin. The superiority margin was set at 2, which has been reported as the minimal clinically important difference in the BBS of patients with post-stroke hemiplegia (Tripepi et al., 2020).

### 2.6 Sample size calculation

PASS 11.0 for Windows was used to estimate the required sample size based on a power calculation to detect between-group differences in the primary outcome measure (BBS) using data from a pilot study conducted with 10 participants (5 per group). We considered two groups and two measurements for primary outcomes to obtain 80% statistical power (1-β error probability), with an α error level probability of 0.05 using superiority test measures. The estimated sample size requirement was 36 patients per group, plus an additional 20% to account for those lost to follow-up, resulting in an estimated sample size requirement of at least 46 patients per group.

## 3 Results

### 3.1 Patients and baseline characteristics

**FIGURE 2:**
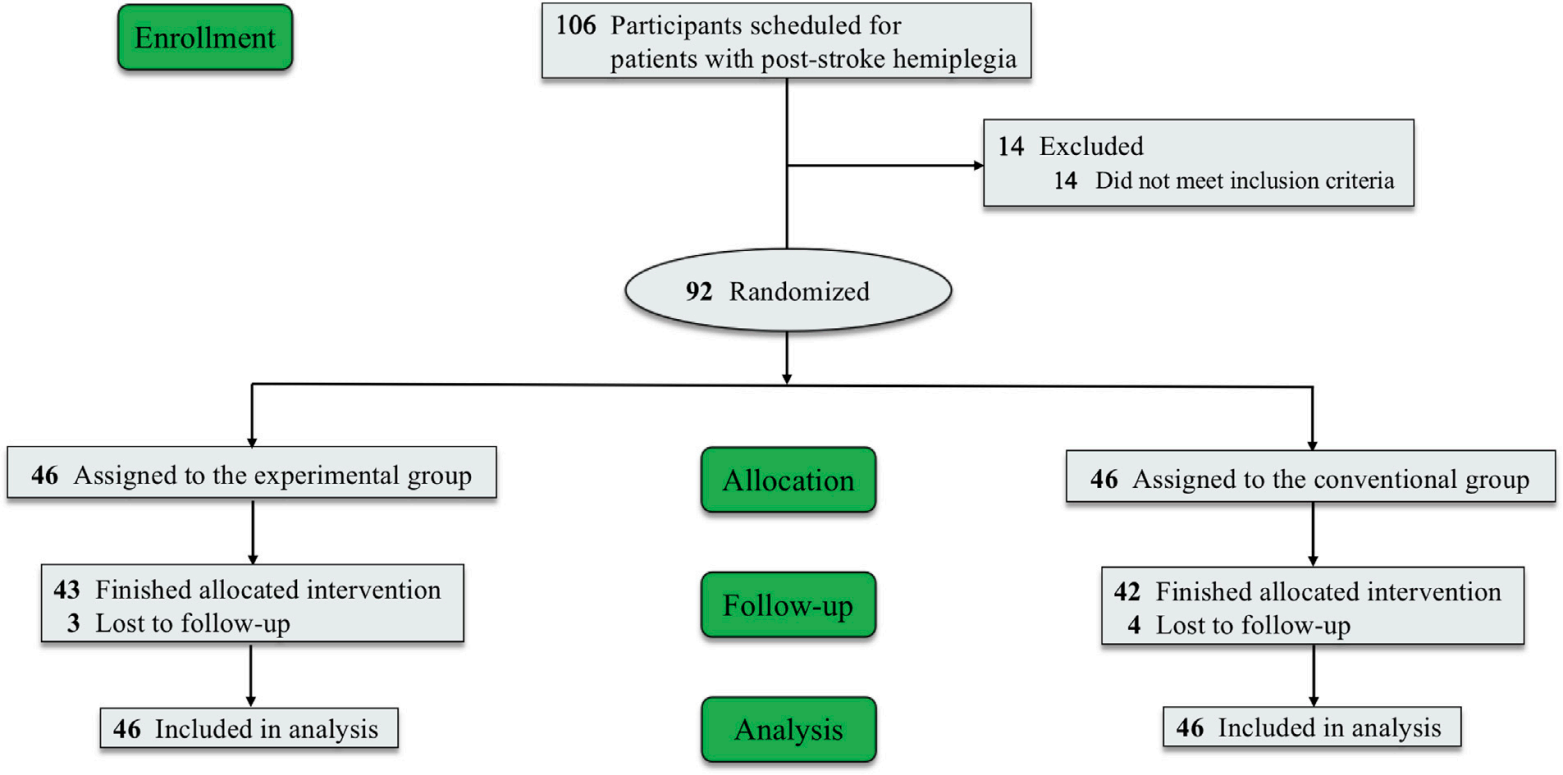
Study flowchart.

A total of 106 patients were identified as patients with post-stroke hemiplegia, among whom 92 patients were enrolled and randomly assigned to the EG (n = 46) and CG (n = 46); 14 patients did not meet the inclusion criteria. Seven (7.6%) patients (three in the EG and four in the CG) withdrew from the study owing to time commitment or personal issues. Among the patients who completed the intervention, 43 were in the EG and 42 in the CG (Figure 2). With the exception of sex, there were no significant differences in the baseline characteristics of the participants between the groups (*P* < 0.05) (Table 2).

**TABLE 2:** Baseline characteristics of all participants.

Baseline characteristic	Experimental group (n = 46)	Conventional group (n = 46)	*P*-value
Age (years)	58.8 ± 10.3	60.8 ± 10.9	0.3690
Sex			0.0444
Male, number (%)	36 (78.3)	26 (56.5)	
Female, number (%)	10 (21.7)	20 (43.5)	
Weight (kg)	67.6 ± 9.7	67.2 ± 10.5	0.8476
Height (cm)	166.3 ± 7.1	165.8 ± 6.9	0.7637
Time from onset of stroke (days)	15.6 ± 7.5	15.1 ± 7.9	1.0000

### 3.2 Effectiveness

**FIGURE 3:**
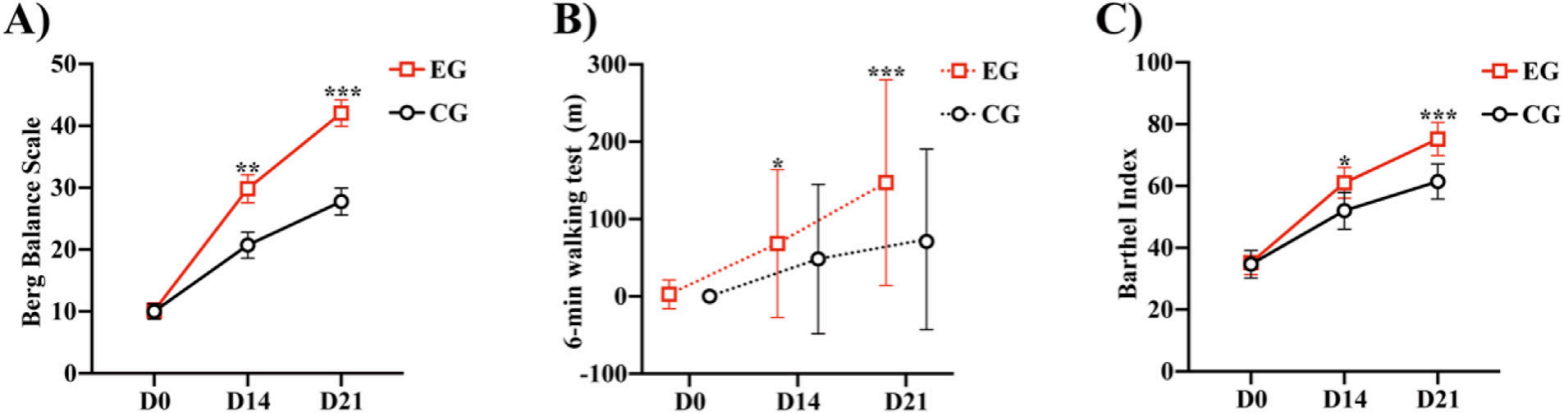
Comparison of the Berg Balance Scale, 6MWT, and Barthel between the two groups: **(A)** Berg Balance Scale of the two groups at different time points. **(B)** 6-min walk test of the two groups at different time points. **(C)** Barthel Index of the two groups at different time points *P < 0.05, **P < 0.01, ***P < 0.0001 vs. CG. Abbreviations: EG, Experimental group; CG, Conventional group.

Significant differences in the BBS score were observed between the two groups at 21 (*P* < 0.0001) and 14 days (*P* < 0.0047) (Table 3; Figure 3A). From before to 21 days after rehabilitation training, the improvement in the BBS score was significantly greater in the EG than in the CG (32.1 ± 12.4 and 17.5 ± 8.6, respectively; *P* < 0.0001). The lower limit of the 95% confidence interval (Cl) in both the groups was greater than 2 (range: 9.96–19.18 and 12.50–20.99, respectively), indicating that the EG had significantly superior therapeutic benefit on balance compared with the CG. These results indicate that compared with routine rehabilitation treatment, assisted unilateral lower limb exoskeleton rehabilitation can restore the balance function of limbs faster and superiorly after stroke hemiplegia.

**TABLE 3:** Comparison of the Berg Balance Scale between the two groups.

Berg balance scale (BBS)	Experimental group (n = 46)	Conventional group (n = 46)	*P*-value	95% CI
D0	10.1 ± 8.5	10.0 ± 8.5	0.9512	—
D14	30.2 ± 15.1	21.1 ± 14.0	0.0047	—
D21	42.0 ± 13.9	27.6 ± 13.7	<0.0001	—
D21-D0	32.1 ± 12.4	17.5 ± 8.6	<0.0001	9.96–19.18

Data are presented as the mean ± standard deviation.

Abbreviations: CI, confidence interval.

**FIGURE 4:**
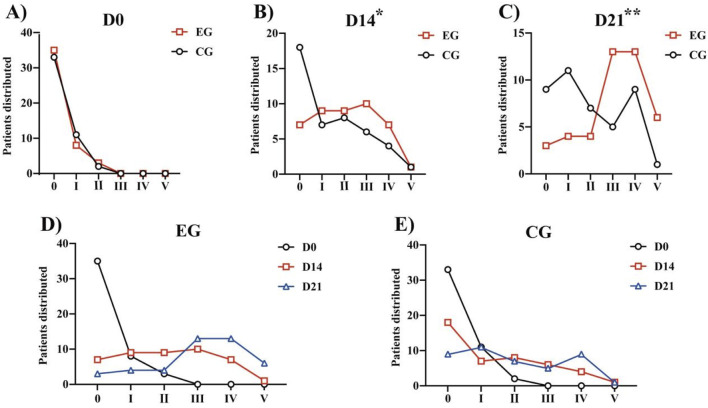
FAC of different grades in patients before treatment and 14 days and 21 days after treatment: **(A)** Distribution of patients with FAC grades at Day 0. **(B)** Distribution of patients with FAC grades at Day 14. **(C)** Distribution of patients with FAC grades at Day 21. *P < 0.05, **P < 0.01 vs. CG. **(D)** Within-group distribution of FAC grades of patients in the EG. **(E)** Within-group distribution of FAC grades of patients in the CG. Abbreviations: FAC, Functional Ambulation Category; EG, Experimental group; CG, Conventional group.

Both the groups demonstrated improvement in the FAC, 6MWT, and Barthel after 21 days of rehabilitation training (Table 4). We calculated the distribution lines of FAC of different grades in patients before and 14 and 21 days after treatment. Before treatment, the EG and CG had roughly the same distribution trend (Figure 4A). Fourteen days after rehabilitation treatment, patients in the CG were mostly rated as grade 0, while those in the EG were >grade 0; the number of patients with high grades was higher in the EG than in the CG (Figure 4B). At 21 days after rehabilitation treatment, most patients in the EG were rated as ≥ grade 3, and the distribution number was much larger than that in the CG (Figure 4C). We also compared the within-group distribution of FAC grades of patients in the EG and CG at different time periods. Moreover, the differences between the EG and CG at 14 (FAC, *P* = 0.0377) and 21 (FAC, *P* = 0.0015) days were statistically significant. With the accumulation of rehabilitation time after receiving the experimental treatment, the gait grade of patients was gradually improved, mainly above grade 3, which was superior to the improvement of patients who received conventional rehabilitation treatment (Figures 4D, E). Likewise, significant differences in the 6MWT (Figure 3B) and Barthel (Figure 3C) were observed between the two groups at 14 days (6 MWT, *P* = 0.0494; Barthel, *P* = 0.0225) and 21 days (6 MWT, *P* = 0.0005; Barthel, *P* = 0.0004).

**TABLE 4:** Comparison of the Functional Ambulation Category, 6-min walk test, and Barthel Index between the two groups.

		Experimental group (n = 46)			Conventional group (n = 46)			*P*-value 14-days	*P*-value 21-days
Time measures		D0	D14	D21	D0	D14	D21		
Functional Ambulation Category (FAC) (Number of patients)	Grade 0	35	7	3	33	18	9	0.0377*	0.0015**
	Grade I	8	9	4	11	7	11		
	Grade II	3	9	4	2	8	7		
	GradeIII	0	10	13	0	6	5		
	Grade IV	0	7	13	0	4	9		
	Grade V	0	1	6	0	1	1		
6-min walking test (6MWT) (m)		0.00 (0.0, 0.0)	24.00 (0.0, 108.0)	120.00 (26.0, 215.0)	0.00 (0.0, 0.0)	0.00 (0.0, 67.25)	0.00 (0.0, 100.0)	0.0494*	0.0005**
Barthel Index (Barthel)		35.2 ± 13.2	61.5 ± 16.3	75.2 ± 17.3	34.7 ± 15.0	52.5 ± 19.7	61.2 ± 18.0	0.0225*	0.0004**

Data are presented as the mean ± standard deviation or median (25th percentile, 75th percentile).

Group design t-test or Wilcoxon signed-rank test was used in the differences of D0, D14, and D21 between groups.

*P < 0.05, **P < 0.01, ***P < 0.0001.

Combined with the BBS results, these data show that regarding balance function, a unilateral lower limb exoskeleton combined with conventional treatment for post-stroke hemiplegia is more effective than conventional rehabilitation treatment. Furthermore, it showed that using a unilateral lower limb exoskeleton combined with conventional treatment can better help patients with hemiplegia after stroke improve gait stability and daily living ability.

### 3.3 Safety

Thirty-eight patients reported AEs: 22 (47.83%) from the EG and 16 (34.78%) from the CG. All AEs were mild and included diarrhea, *clostridium difficile* infection, abnormal liver function, left wrist fracture, and swelling and pain in gingival and forearm soft tissue. Only one (1.1%) AE (waist soreness) was judged as potentially device related, while the others were unrelated to the device (Table 5). No significant difference in the proportion of patients with AEs was observed between the groups (*P* = 0.204).

**TABLE 5:** Group differences in safety analysis outcomes.

Safety analysis outcome	Experimental group (n = 46)	Conventional group (n = 46)	*P*-value
AEs, n (%)	22 (47.83)	16 (34.78)	0.204
SAEs, n (%)	0 (0)	0 (0)	—
Device-related AEs, n (%)	1 (2.17)	0 (0)	—
Device-related SAEs, n (%)	0 (0)	0 (0)	—

Abbreviations: AE, adverse event; SAE, severe adverse event.

Only one AE was considered to be possibly caused by the device.

## 4 Discussion

Stroke can cause damage to the upper motor neurons of the patient, causing the lower central motor reflex that should be suppressed to become active (Emos and Agarwal, 2023). This causes a series of spinal cord level movement patterns, such as an abnormal increase in muscle tone on the affected site, abnormal joint responses and co-movement, disruption of reciprocal inhibition, and abnormal intermuscular coordination, which are manifested as poor balance, weak muscle strength, poor control, and abnormal movement patterns (Qi et al., 2024). According to the theory of rehabilitation, active rehabilitation, regular exercise, and sensory stimulation are conducive to the plasticity of the CNS (Shen, 2022). Active rehabilitation can promote the functional recovery of the tissue around the injured brain, compensate for the healthy brain cells, accelerate the establishment of collateral circulation, and promote the functional recovery, together forming the motor relearning program. This treatment approach was proposed in the early 1980s (Winterbottom and Nilsen, 2024). Active motor relearning therapy is recommended for stroke rehabilitation. It is a process of relearning or retraining for the recovery of motor function after CNS injury. Based on theories of biomechanics, sports science, and cognitive psychology, it emphasizes the practicability of motor function and the active participation of patients (Ghrouz et al., 2024).

Based on the principle of brain and nerve plasticity, stroke patients with hemiplegia can receive repeated stimulation of the remodeling cerebral cortex by participating in highly repetitive, intensive, and correct balance and movement patterns and in time, retain this correct balance and movement pattern in the brain (Regele-Blasco and Palmer, 2024). LiteStepper^®^ does this by using kinematic, dynamic, and mechanical redundant sensing technology to accurately perceive the patient’s motion intention and gait characteristic parameters, copy the balance and gait data of the healthy side leg, and provide reference for the movement of the hemiplegic side leg. The healthy side leads the affected side to perform bilateral joint gait and balance training, thus forming a “symmetrical” interactive gait. This is more conducive to the guidance of the therapist (Sorkhabadi et al., 2023). Using this approach, the problem of gait imbalance and asymmetry in rehabilitation training was solved successfully, and accurate and personalized rehabilitation training was realized. The results of the present study show that the device can improve the patient’s participation in treatment, promote the recovery of patients’ balance and motor abilities, and aid in the return of relatively normal life abilities. This also shows that the comprehensive rehabilitation treatment program based on the principle of active movement relearning therapy is more conducive to promoting the recovery of patients’ balance, motor function, and activities of daily living and enabling efficient rehabilitation.

The first condition for normal walking is balance, which is a crucial factor in the ability to walk independently. The BBS, proposed by Berg et al., in 1989 and widely used in clinical practice, includes 14 different balance items such as standing, bending, turning, and moving, to assess a patient’s dynamic and static balance (Miranda-Cantellops and Tiu, 2024). The efficacy of previous robot-assisted therapy for balance function has been evaluated using the BBS (Wang et al., 2021; Peláez-Vélez et al., 2023; Shao et al., 2023). These studies show that the restoration of balance function is the primary goal of rehabilitation for hemiplegic patients (Calisgan and Talu, 2024). It is also key to achieving optimal gait function in patients with hemiplegia after a stroke (Yang et al., 2023; Ayvat et al., 2024). Therefore, in this study, we used the BBS as the main outcome indicator.

Generally, the human leg structure consists of seven degrees of freedom per leg. The ULLE used in this study features five degrees of freedom at the ankle, knee, and hip joints. The joint motors are controlled by the system’s main unit, which learns normal gait and balance functions, driving the patient’s affected leg in a cyclic and rhythmic motion. This is more complex and flexible than simpler rehabilitation devices such as ankle orthoses and adaptive gait trainers. Studies have reported that earlier and more intensive limb activity post-stroke contributes to faster recovery of balance and motor functions (Oliveira et al., 2024). Our study showed that compared to the conventional rehabilitation group, the balance ability of patients in EG was significantly improved. The balance score of EG was nearly twice as high as that of CG. By the 21st day of the experiment, the walking ability in EG was mainly grade 3 or above, while more than half of the CG was below grade 3. Additionally, the FAC, 6MWT, and Barthel indicated that the daily activity ability of the EG was better than that of the CG. These results suggest that LiteStepper^®^ has a significant auxiliary effect on balance and motor function rehabilitation in patients with hemiplegia after stroke. During the entire study, 38 cases of AEs were found, all of which were mild. Among them, diarrhea and abnormal liver function were adverse reactions of the digestive system. Left wrist fractures, gum pain, and forearm soft tissue swelling and pain were related to the motor and nervous systems. These were found to be unrelated to the instruments after cause analysis, and we provided symptomatic treatment. The fractures were associated with the subject’s osteoporosis and falls, gum pain resulted from the subject’s pre-existing condition, and soft tissue swelling and pain in the forearm were associated with skeletal muscle stiffness after a stroke. There was one subject with lower back pain, which was closely related to the rehabilitation with the exoskeleton lower limb robot. After the patient was given sufficient rest, the experiment continued once the pain subsided, and the patient was instructed on performing correct skeletal muscle exercises.

Compared with existing clinical rehabilitation equipment products, the LiteStepper^®^ ULLE has the advantages of switching from traditional passive training to intelligent active training; the patient’s brain controls its healthy limb walking to drive the affected limb to form interactive walking, and the awakening and use of the patient’s active rehabilitation consciousness can maximize the rehabilitation efficiency of the brain (Marín-Medina et al., 2024). Additionally, from programmed control to personalized learning control, the gait control of the affected limb of LiteStepper^®^ ULLE is based on the perception of the movement intention and gait characteristics of the limbs, and the object of learning is no longer “program” or “others” but rather the patient’s own limbs, which help the patients carry out personalized rehabilitation. The personality-optimized movement pattern of the affected limb can help the affected limb systematically recover its neural control function with the correct movement pattern during walking training and effectively reduce the disability rate caused by abnormal gait. Finally, from peripheral nerve stimulation to simultaneous central and peripheral nerve stimulation (Kern et al., 2023), LiteStepper^®^ LiteStepper^®^ ULLE stimulates the patient’s active rehabilitation consciousness; by this means, the patient’s CNS and PNS are trained concurrently, contributing to the efficient rehabilitation of the patient’s overall nervous system.

### 4.1 Limitations of this study

This study, despite its merits, was constrained by several limitations that merit attention. Firstly, the lack of follow-up observations hindered our ability to comprehensively assess the long-term impacts of BBS-assisted training on patients’ limb function and overall recovery. This limited our understanding of the device’s sustained effectiveness. Secondly, the relatively modest sample size employed in this study restricts our capacity to conduct stratified analyses that could potentially uncover nuanced differences in treatment responses across various subgroups. This constraint prevented us from drawing more nuanced conclusions regarding the generalizability and effectiveness of the intervention. Thirdly, the absence of comprehensive gait analysis represented a notable gap in our understanding of how BBS-training specifically influences gait dynamics in patients with hemiplegia. Such analysis would have provided a deeper, more nuanced insight into the intervention’s impact on functional mobility and gait patterns. Recognizing these limitations, future research endeavors should aim to incorporate long-term follow-ups, larger sample sizes, and comprehensive gait assessments to enhance the rigor and comprehensiveness of their findings.

### 4.2 Conclusion

Our study evaluated the efficacy of LiteStepper^®^ ULLE, in conjunction with conventional therapy, on balance function training among patients with post-stroke hemiplegia. Ninety-two patients in the late stage of stroke were randomly assigned to either the EG or the CG. The EG received a 21-day intervention combining LiteStepper^®^ ULLE with routine treatment, whereas the CG underwent a standard daily rehabilitation program for the same duration.

The study employed the BBS, FAC, 6 MWT, and Barthel as assessment tools to comprehensively evaluate balance function, walking ability, and activities of daily living (ADL) independence before and after rehabilitation training in both groups. The results demonstrated that at the 14th and 21st day evaluations, the BBS scores of the EG were significantly higher than those of the CG, with a notably greater improvement margin observed in the EG. Additionally, the EG exhibited significantly superior improvements in FAC, 6 MWT, and Barthel Index scores compared to the CG. Notably, only one adverse event related to LiteStepper^®^ ULLE was reported throughout the study period, underscoring the high safety profile of this device when combined with routine balance training.

In summary, the integration of LiteStepper^®^ ULLE with conventional therapy has shown remarkable advantages in enhancing balance function, walking ability, and ADL independence among post-stroke hemiplegia patients. Furthermore, this therapeutic approach demonstrates a high level of safety. This finding introduces a novel and effective means for rehabilitation in stroke survivors, thereby possessing significant clinical implications and value.

## Data Availability

The raw data supporting the conclusions of this article will be made available by the authors, without undue reservation.
