# Supplementary material for: Efficacy and safety of using a unilateral lower limb exoskeleton combined with conventional treatment in post-stroke rehabilitation: a randomized controlled trial
Source: Front Bioeng Biotechnol. 2024 Sep 18;12:1441986. doi: 10.3389/fbioe.2024.1441986 (PMC11445659; doi:10.3389/fbioe.2024.1441986)
Supplement: Supplementary file 1 [file DataSheet1.docx]

Supplementary Material

# Supplementary Figures

**
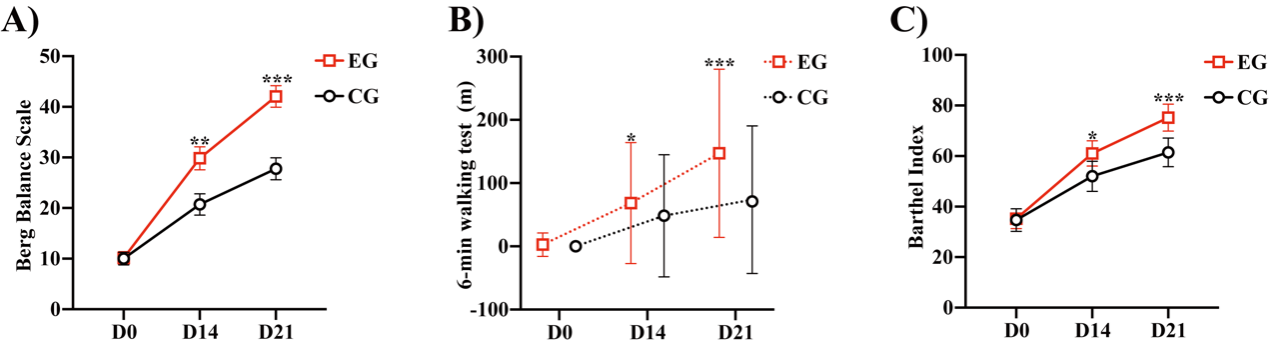
**

**Supplementary Figure 1.** Comparison of the Berg Balance Scale, 6MWT, and Barthel between the two groups

A) Berg Balance Scale of the two groups at different time points. B) 6-min walk test of the two groups at different time points. C) Barthel Index of the two groups at different time points **P* < 0.05, ***P* < 0.01, ****P* < 0.0001 vs. CG.

Abbreviations: EG, Experimental group; CG, Conventional group.


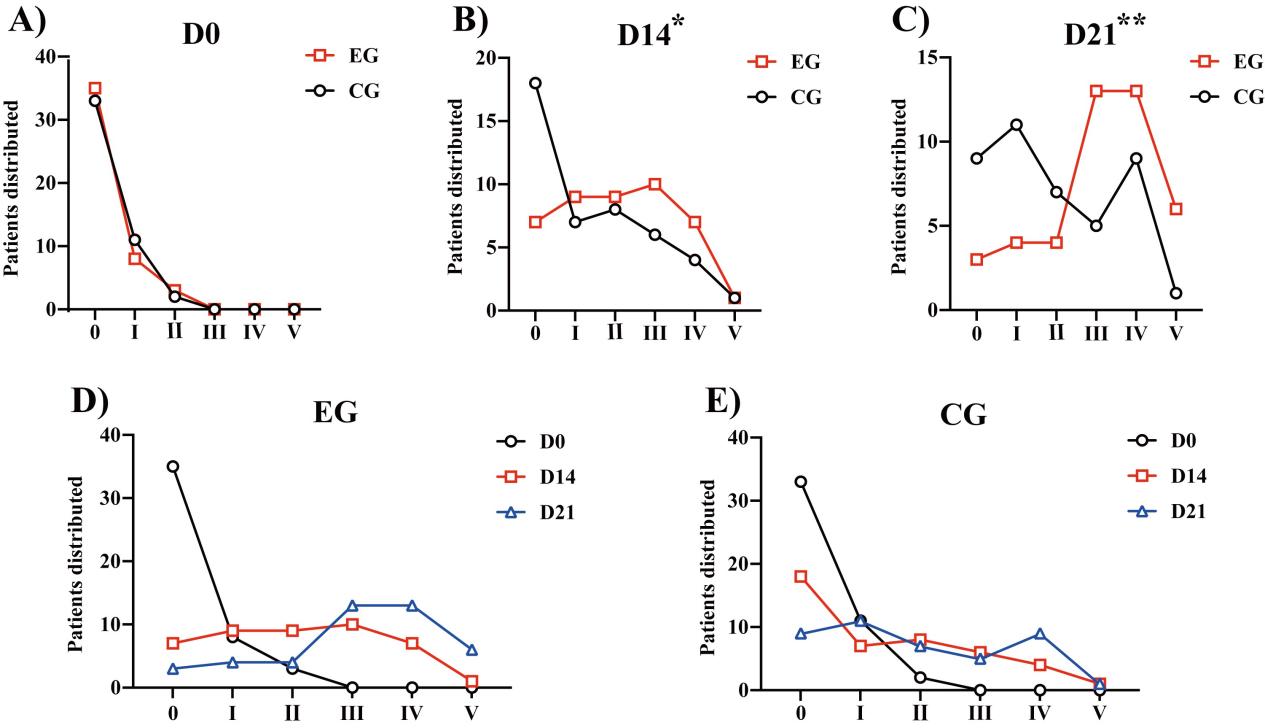


**Supplementary Figure 2.** FAC of different grades in patients before treatment and 14 days and 21 days after treatment

A) Distribution of patients with FAC grades at Day 0. B) Distribution of patients with FAC grades at Day 14. C) Distribution of patients with FAC grades at Day 21. **P* < 0.05, ***P* < 0.01 vs. CG. D) Within-group distribution of FAC grades of patients in the EG. E) Within-group distribution of FAC grades of patients in the CG.

Abbreviations: FAC, Functional Ambulation Category; EG, Experimental group; CG, Conventional group;
